# Editorial: Chronic Illness and Ageing in China

**DOI:** 10.3389/fpubh.2020.00104

**Published:** 2020-04-09

**Authors:** Shane Andrew Thomas, Zeqi Qiu, Anna Chapman, Shuo Liu, Colette Joy Browning

**Affiliations:** ^1^Research School in Population Health, Australian National University, Canberra, BC, Australia; ^2^International Primary Health Care Research Institute, Shenzhen, China; ^3^Department of Sociology, Peking University, Beijing, China; ^4^School of Nursing and Midwifery and Centre for Quality and Patient Safety Research, Deakin University, Burwood, VIC, Australia; ^5^Key Laboratory of Carcinogenesis and Translational Research, Beijing Office for Cancer Prevention and Control, Peking University Cancer Hospital & Institute, Beijing, China; ^6^School of Nursing and Healthcare Professions, Federation University, Ballarat, VIC, Australia

**Keywords:** chronic illness, aging, China, behavior change, health, well-being

This Research Topic focussed on chronic illness and aging in China. China's population is aging and chronic illnesses are increasingly prevalent (1–4). China's population life expectancy has increased by 17 years since 1970. From 2010 to 2040, it is predicted that the proportion of people aged 60 years plus in China will increase from 12.4% (168 million) to 28% (402 million) (1). There is a progressive shift in the burden of disease to chronic Non-Communicable Diseases (NCDs). Almost 80% of deaths in China in people aged 60 years are from chronic NCDs (5), the most burdensome being Ischemic Heart Disease, Stroke, Chronic Obstructive Pulmonary Disease (COPD), and Type 2 Diabetes. Behavioral risks including smoking, alcohol consumption, sedentary behavior and poor dietary intake contribute significantly to these conditions.

China has been preparing for the dramatic aging of its population and the health transition from communicable diseases to NCDs. In this Research Topic 40 researchers from throughout China and invited international colleagues, examined a range of topics impacting on the health and well-being of older people living in China. These included nutrition, smoking, cognitive impairment, cardiovascular health, sensory loss and diabetes as well as how to prepare the healthcare workforce to provide effective services for older adults. A range of methodologies and article types comprise the Research Topic: a case study, national surveys, a randomized controlled trial, qualitative interviews, a focussed review and predictive studies using multiple regression. These papers focus on preparing the world's most populous country to deal effectively with the health of its seniors and be positioned to share lessons learned with others.

Two papers in this issue focussed on general practitioner training. Rao et al. reported curriculum development research to prepare Chinese GPs for increased chronic disease prevalence. The curriculum was trialed with 600 GP trainees. Sun et al. analyzed the epidemiology of Chinese chronic illness and their associated risk factors. They argued that upgrading of Chinese General Practitioner skills is necessary to meet contemporary and future health needs of China.

Heine et al.; Heine et al. used the China Health and Retirement Survey (CHARLs) Wave 2, 2013 data for people aged 60 years and over (*n* = 8,268). The authors concluded that sensory loss especially dual sensory loss was significantly and positively associated with advanced age, having difficulty in any Activities of Daily Living (ADL) or Instrumental Activities of Daily Living (IADL) and experiencing depression and less life satisfaction. They also found that sensory loss is a significant health issue for older Chinese people that strongly affects their social participation that needs to be reflected in practitioner training and services. This is an area that requires increased resources and attention.

Browning et al. reported a qualitative study of older Chinese people concerning their experiences of food and its cultural impacts and importance for health and well-being in China and confirmed the high cultural importance of food for older Chinese. It was found that social and economic lifespan experiences continued to strongly impact on the food and eating attitudes and practices of older Chinese people. These experiences differed markedly from younger Chinese suggesting that health promotion messaging involving food and nutrition must be targeted to maximize its effectiveness amongst older Chinese.

Two studies in this issue examined the association between cardiovascular health and diet and cognitive function in older Chinese. Gildner et al. reported analyses of the WHO Study on global Aging and adult health (SAGE) Wave 1 (*n* = 11,295). Seven Cardiovascular Health (CVH) factors were classified as “ideal” or “not ideal” including smoking and drinking frequency, Body Mass Index, physical activity level, blood pressure, diet, and self-reported anxiety. It was found that “ideal” CVH factors predicted higher cognitive performance. This study suggested that early detection and controlling modifiable CVH risks may protect aging individuals in China from cognitive decline, a major benefit.

Xu et al. analyzed a 10-year sequence of participants aged 55+ (*n* = 4,847) from the China Health and Nutrition survey. Linear mixed statistical models were used to investigate the association between dietary patterns, hypertension, and cognitive function. Three dietary patterns were identified “Traditional Chinese,” “Protein-rich,” and “Starch-rich.” The Protein-rich dietary pattern was associated with higher cognitive global and verbal memory scores, while the starch-rich dietary pattern (high intake of salted vegetable and legumes) was associated with lower cognitive global and verbal memory scores as was hypertension. The study reinforced the importance of diet and management of hypertension for preventing cognitive decline.

Yiengprugsawan and Browning reported a focussed review of the link between NCDs and Cognitive Impairment and Mild Cognitive Impairment (CI/MCI) through common risk factors. They argued that the identification of risk factors is critical for the effective prevention and management of chronic conditions including cognitive impairment in China and globally. The review also identified the importance of primary health care services in reducing behavioral risk factors. The authors argued that addressing shared determinants and pathways was important in the design of public health and primary health care services in China. The results of these papers paint a clear picture of the potential benefits of evidence-based interventions in maximizing the cognitive health in China.

Hou et al. used data from CHARLs to study the smoking behaviors of Chinese rural-to-urban migrants, urban-to-urban migrants, rural return migrants, and urban return migrants. Smoking is a major chronic illness risk factor in China especially amongst older men. Strong associations between migration status and later-life smoking behaviors in China were discovered and that these associations varied greatly according to different migration status. These findings provide important data to inform smoking cessation programs in China.

Chapman et al. reported a diabetes management Randomized Controlled Trial conducted in Beijing (*n* = 726). “Control” participants received usual care according to the Chinese Guideline for Diabetes Prevention and Management. “Intervention” participants received Motivational Interviewing (MI) health coaching plus usual care. The participants displayed significant within-group HbA1c improvements at 18-months. The findings suggested that there is benefit in training Chinese clinicians in coaching and behavior change techniques.

We consider that to meet the health care needs of China that the outcomes of ongoing and detailed epidemiological analyses and research must be directly reflected in health system design and practitioner training. The papers in this Research Topic provide important guidance. Benchmarking against international standards is useful as well as understanding the unique and changing needs of older people in China. While ongoing research is warranted, the studies reported in this Research Topic inform pragmatic changes to the prevention and treatment of chronic conditions among older Chinese and training of the healthcare workforce.

## Author Contributions

All authors listed have made a substantial, direct and intellectual contribution to the work, and approved it for publication.

### Conflict of Interest

The authors declare that the research was conducted in the absence of any commercial or financial relationships that could be construed as a potential conflict of interest.
